# Turbo-FLASH Based Arterial Spin Labeled Perfusion MRI at 7 T

**DOI:** 10.1371/journal.pone.0066612

**Published:** 2013-06-20

**Authors:** Zhentao Zuo, Rui Wang, Yan Zhuo, Rong Xue, Keith S. St. Lawrence, Danny J. J. Wang

**Affiliations:** 1 State Key Laboratory of Brain and Cognitive Science, Institute of Biophysics, Chinese Academy of Sciences, Beijing, China; 2 UCLA-Beijing Joint Center for Advanced Brain Imaging, Beijing, China and Los Angeles, California, United States of America; 3 Lawson Health Research Institute, London, Ontario, Canada; 4 Department of Neurology, University of California Los Angeles, Los Angeles, California, United States of America; University of California San Francisco, United States of America

## Abstract

Motivations of arterial spin labeling (ASL) at ultrahigh magnetic fields include prolonged blood *T_1_* and greater signal-to-noise ratio (SNR). However, increased B_0_ and B_1_ inhomogeneities and increased specific absorption ratio (SAR) challenge practical ASL implementations. In this study, Turbo-FLASH (Fast Low Angle Shot) based pulsed and pseudo-continuous ASL sequences were performed at 7T, by taking advantage of the relatively low SAR and short TE of Turbo-FLASH that minimizes susceptibility artifacts. Consistent with theoretical predictions, the experimental data showed that Turbo-FLASH based ASL yielded approximately 4 times SNR gain at 7T compared to 3T. High quality perfusion images were obtained with an in-plane spatial resolution of 0.85×1.7 mm^2^. A further functional MRI study of motor cortex activation precisely located the primary motor cortex to the precentral gyrus, with the same high spatial resolution. Finally, functional connectivity between left and right motor cortices as well as supplemental motor area were demonstrated using resting state perfusion images. Turbo-FLASH based ASL is a promising approach for perfusion imaging at 7T, which could provide novel approaches to high spatiotemporal resolution fMRI and to investigate the functional connectivity of brain networks at ultrahigh field.

## Introduction

During the past two decades, arterial spin labeled (ASL) perfusion MRI has been developed into a class of noninvasive methods for direct measurement of cerebral blood flow (CBF). It has found a broad range of applications in both basic and clinical neuroscience [1], [2]. The drawbacks of ASL, however, include the relatively small fractional signal of labeled arterial blood (∼1%) as well as the transit artifact resulting from intravascular labeled blood yet to reach brain tissue by the time of image acquisition [1], [3]. For instance, ASL perfusion imaging in patients with ischemic stroke has been challenging, since the delivery of labeled blood to capillaries and brain tissue can be severely delayed. While alternative spin labeling strategies such as velocity selective labeling [4] have been proposed to reduce the sensitivity of ASL to delayed arterial transit effects, these methods often suffer from reduced signal strengths compared to standard ASL approaches.

The availability of magnets with high and ultrahigh field strengths (≥4.0 T) offers a potentially interesting approach to circumvent the limitations of ASL [5]. First, the signal-to-noise ratio (SNR) is proportional to the main field strength. Second, the “tracer half life” as determined by the longitudinal relaxation time (*T_1_*) of labeled blood water is prolonged at higher magnetic fields, which follows approximately a cube root increase with the resonance frequency (ω^0.3^) [5], [6], [7], [8]. Lastly, prolonged tracer relaxation times and increased SNR should allow the use of relatively long post-labeling delay times to counteract delayed arterial transit effects in ASL. To date, implementations of pulsed [5], [9] and pseudo-continuous ASL (pCASL) for human studies [6], [10], [11] have been carried out at 4.0 and 7.0T. While a general benefit of SNR gain has been demonstrated, the implementation of ASL at ultrahigh fields presents several challenges.

One major challenge is that inhomogeneities in B_0_ and B_1_ fields become more prominent at higher field strengths, leading to compromised label efficiency. In particular, past studies have shown that corrections of B_0_ and B_1_ fields may have to be performed for effective implementation of pCASL at 7T [6]. In addition, transverse relaxation times (*T_2_* and T_2_^*^) are shortened due to stronger susceptibility effects at higher magnetic fields [12]. This effect of faster *T_2_*/T_2_^*^ decay may discount the ASL signal gain through prolonged *T_1_* relaxation at higher fields and may complicate CBF quantification [13]. Most existing ASL studies have used gradient-echo echo-planar imaging (EPI) as the readout sequence which suffers from susceptibility effects (signal loss and image distortions) that are more pronounced at ultrahigh field strengths [14], [15]. Lastly, the specific absorption ratio (SAR) of RF power increases with the square of resonance frequency, limiting the duration and number of labeling pulses that can be applied at ultrahigh fields.

Turbo-FLASH (Fast Low Angle Shot) is a promising approach for fast imaging at ultrahigh fields due to its relatively low SAR and short TE that minimizes susceptibility artifacts [16], [17], [18]. In addition to its use for structural and susceptibility MRI at 7T, Turbo-FLASH may be suitable for ASL perfusion imaging as the longitudinal magnetization of the label can be well preserved by the RF pulse train with small flip angles. On the other hand, the short TE (a few ms) of Turbo-FLASH ensures minimal decay of the label through transverse relaxation [19]. The goal of the present work was therefore to implement and optimize Turbo-FLASH based pulsed ASL (PASL) and pCASL at 7T to explore and maximize the SNR gain of ultrahigh field ASL. As a proof-of-concept study, perfusion imaging was performed on the motor cortex where B_0_ and B_1_ fields are relatively homogeneous. Perfusion based functional MRI (fMRI) of motor cortex activation was further performed to demonstrate the sensivitity of Turbo-FLASH based ASL at 7.0T to neural activation. Finally, functional connectivity analysis of motor cortices was performed on resting state perfusion image series collected at 7.0T.

### Theory

**Figure 1:**
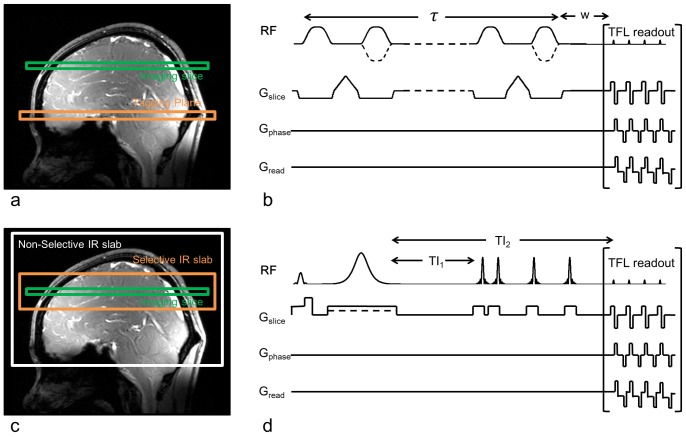
Schemes of pulse sequence for PASL and pCASL. (a) Location of tagging and control plane of pCASL, green is the imaging plane and orange is tagging or control plane, (b) Pulse sequence diagram of Turbo-FLASH based pCASL, w is the post-labeling delay, (c) Location of the selective inversion slab or non-selective inversion slab, and the imaging plane, (d) Pulse sequence diagram of Turbo-FLASH based PASL, TI_1_ is the tagging duration and TI_2_ is the total duration of tagging and post-labeling delay. A series of inferior saturation pulses were applied after TI1 for complete spoil of the residual tagging bolus.

The theoretical assumptions were based on the two ASL pulse sequences employed in the experiment, namely FAIR with QUIPSS II type [20], [21] inferior saturation for pulsed ASL (PASL) and pCASL with balanced gradients between label and control [22] (see Figure 1 for details of pulse sequences and labeling schemes). For the sake of simplicity, a standard single-compartment model was adopted for theoretical calculation which assumes that labeled blood water stays in the vasculature rather than exchanging into tissue [23]. The effect of water exchange between capillaries and tissue is discussed below.

The standard PASL signal can be modeled by [23], [24]:
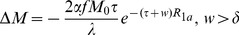
(1)where the difference perfusion signal, 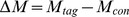, *M_tag_/M_con_* is the longitudinal magnetization following the tagging or control pulse but before the first excitation RF pulse respectively, *R_1a_* is the longitudinal relaxation rate of blood, *α* is the tagging efficiency, *τ* is the duration of tagging bolus (or *TI*_1_), *w* (or *TI*) is the post-labeling delay time, *δ* is the arterial transit time and *λ* ( = 0.9 g/ml) is the blood/tissue water partition coefficient. The tag and control signals, *S_tag_*/*S_con_*_,_ acquired by the Turbo-FLASH sequence are [25]:
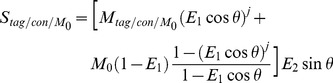
(2)where 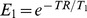, and 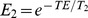, M0 is the equilibrium magnetization, θ is the flip angle of Turbo-FLASH, j is the number of k-space lines acquired by the FLASH readout. The Turbo-FLASH based PASL signal, *Δ*S, can be calculated by:



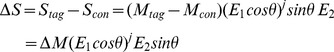
(3)By substituting Eqs. (1) and (2) into Eq. (3), Turbo-FLASH based PASL signals can be modeled by:
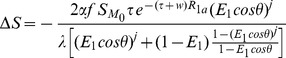
(4)


Similarly, the standard pCASL signal can be calculated by [26]:
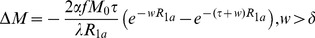
(5)


By substituting Eqs. (2) and (5) into Eq. (3), Turbo-FLASH based pCASL signals can be modeled by:
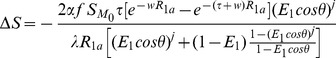
(6)


## Methods

### Theoretical Calculation

Theoretical calculation was performed using custom MATLAB (MathWorks, Inc., MA, USA) programs. Turbo-FLASH based PASL and pCASL signals as a function of field strength were calculated based on Eqs. (4) and (6) respectively. Blood *T_1_* was assumed to follow an approximate cube root increase with the resonance frequency (ω^0.3^) [5], [6], [27]. A blood *T_1_* of ∼2290 ms has been reported at 7T [7]. Due to the use of a short TE of 1.36 ms, the effect of transverse relaxation times on Turbo-FLASH based ASL signals was small (<2% change between 3T and 7T) and was ignored [28]. Other assumed parameters were: TR (between 2 excitation pulses) = 3.11 ms, labeling efficiency = 0.95 and 0.8 for PASL and pCASL [22], flip angle = 7° (the Turbo-FLASH signal reaches maximum with the flip angle of 7° at 7T based on simulation).

### Imaging Sequences

Imaging was performed on a 7T whole-body Magnetom system (Seimens Medical Systems, Erlangen, Germany) with 1CP transmit/24 channel receiver head coil (Nova Medical, Cambridge, MA, USA), and a 3T whole-body TIM Trio system with the default 12-ch reciever coil (Siemens Medical Systems, Erlangen, Germany). The pCASL sequence used balanced gradients between tag and control acquisitions (Fig. 1a) [22]. The PASL sequence used a modified version of the FAIR technique [21], in which a series of saturation pulses was applied at 700 ms after the label or slice-selective inversion, similar to QUIPSS II (Fig. 1d). Detailed parameters for labeling pulses of pCASL and PASL can be found in [22]. A standard Turbo-FLASH readout was applied at the post-labeling delay time following labeling pulses of PASL or pCASL, respectively. Two experiments were carried out at both 3T and 7T for comparison: 1) resting state pCASL and PASL scans with different spatial resolution; and 2) fMRI of motor cortex activation using PASL along with functional connectivity analysis. The global SAR of the Turbo-FLASH based PASL and pCASL sequence at 7T was below the FDA limit of 4 W/kg in a gram of tissue, as calculated and monitored by vendor supplied programs.

### Exp. 1 Resting-State PCASL and PASL

Five healthy subjects (23–27 years, 3 males) underwent MRI scanning at both 3T and 7T. All experiments were performed according to the principles expressed in the Declaration of Helsinki and had approval from the Human Reasearch Ethics committee of the Institute of Biophysics, Chinese Academy of Sciences. All participants provided written informed consent for the collection of data and subsequent analysis. Acquisition parameters for Turbo-FLASH based pCASL were: FOV = 220×220 mm^2^, TE = 1.36 ms, bandwidth = 490 Hz/pixel, slice thickness = 5 mm, matrix = 128×64, 128×128, 256×128 for 7T, and 64×64, 128×64, 128×128 for 3T, post-labeling delay = 1000 ms, label duration = 700 ms, TR (between label and control acquisitions) = 3000 ms which was adjusted according to SAR limitations. A single slice through the motor cortex was imaged and the label offset was 4.5 cm for pCASL. Thirty pairs of control and label images were acquired for each spatial resolution in each subject at 3T and 7T respectively.

The same 5 subjects underwent resting PASL scans and acquisition parameters were: FOV = 220×220 mm^2^, TE = 1.36 ms, bandwidth = 490 Hz/pixel, slice thickness = 5 mm, matrix = 128×64 at 7T and 64×64 at 3T, post-labeling delay = 1000 ms, tagging pulse duration = 15 ms, TI_1_ = 700 ms, TR = 4000 ms. The selective inversion band was 4 cm for a single slice through the motor cortex. Thirty pairs of control and label images were acquired for each post-labeling delay in each subject at 3T and 7T respectively.

### Exp. 2 fMRI of Motor Cortex Activation and Functional Connectivity using PASL

fMRI experiments with sensorimotor cortex stimulation were performed on the same five healthy subjects at both 3T and 7T using PASL. Acquisition parameters were: TR = 4 s, post-labeling delay = 1000 ms, matrix = 128×64, 128×128 and 256×128 at 7T and 64×64, 128×64 and 128×128 at 3T. The rest parameters were the same as those of resting-state PASL. An alternating finger tapping paradigm (6 cycles of 24 s left hand and 24 s right hand, with an 8 s resting gap) was applied, and each fMRI scan took 6.4 min. In addition, fMRI experiments were performed using a multi-slice PASL sequence on 3 healthy subjects (21–27 years, 2 males) at 7T. The protocol was the same as for the single slice PASL study except that 7 slices with the thickness of 3.5 mm (with 0.735 mm gap) were acquired, TR = 4500 ms and the selective inversion band was 5.5 cm. Multi-slice resting-state PASL scans were then performed with the matrix size of 128×64, 128×128 and 256×128 respectively at 7T. Each of the resting state PASL scans took 10 min with 75 pairs of control and label acquisitions, while the subjects were instructed to rest quietly with eyes closed.

### Phantom Study

In order to compare the coil sensitivities at 3T and 7T, a vendor supplied oil phantom was scanned at both 3T and 7T using Nova Medical volume transmit and 24-ch receiver coil, and the default 12-ch receiver coil, respectively. A proton density (PD) image was acquired using a turbo-spin echo (TSE) sequence (TR = 3000 ms, TE = 17 ms, FA = 120°, FOV = 220×220 mm^2^, matrix size = 320×320, slice thickness = 5 mm) at both 3T and 7T.

### Data Processing

Original images were motion corrected using a six-parameter, rigid-body, least-squares realignment by SPM8 (Wellcome Trust Centre for Neuroimaging, UCL, London, UK). Resting-state PASL and pCASL signals were obtained by pairwise magnitude subtraction between control and label images in each scan, followed by averaging to produce mean *ΔM* images. The SNR was calculated by dividing the average intensity of *ΔM* signals in a region of interest (ROI) within the brain by the standard deviation (SD) of noise in an ROI outside of the brain. The foreground ROI covered most parts of the brain, excluding the veins, skull and cerebrospinal fluid (CSF). The ROI for background noise was carefully chosen to avoid any region presenting apparent unfolding or ghosting artifact. The temporal SNR was calculated as the mean *ΔM* intensity divided by the SD of the *ΔM* image series. CBF values were calculated based on Eq. (4) for PASL and Eq. (6) for pCASL across brain voxels.

For functional perfusion imaging, original images were corrected for motion, and smoothed in space with a 3D Gaussian kernel that was twice of the pixel size, followed by CBF calculation. Statistical analysis was performed on the preprocessed image series using a general linear model (GLM). The effect of interest in the GLM design matrix comprised of a simple boxcar waveform defining different stimulus conditions. The significant activation level was set to P<0.001 with a cluster size >10 voxels. Mean perfusion signal changes were derived from the activated clusters, and compared using repeated measures ANOVA of the SPSS 19.0 software package (SPSS, Inc., Chicago, IL, USA). The detected activation region in multi-slice PASL fMRI scans was used as the seed region for functional connectivity analyses. Resting-state PASL data were realigned, corrected for time lag between slices, followed by linear detrending and temporal filtering with a band-pass filter between 0.01 and 0.08 Hz. Functional connectivity maps were generated by calculating Pearson correlation coefficients between perfusion time courses of each brain pixel with that of the seed ROI. Pearson correlation coefficients were then converted to Z statistics with Fisher’s z transformation.

## Results

### Theoretical Calculation

**Figure 2:**
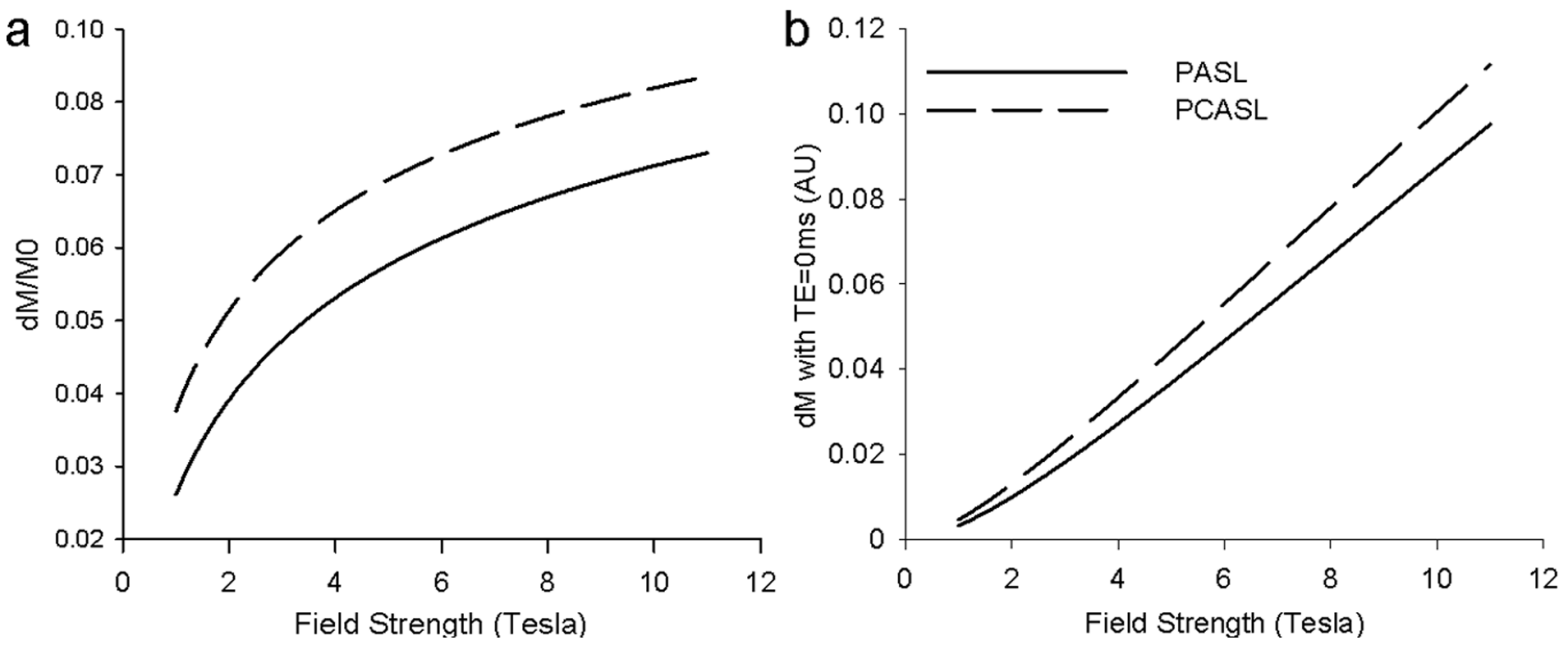
Theoretical Turbo-FLASH based ASL *Δ* *M* signal as a function of main field strength. (a) Fractional *Δ* *M* signal change with field strength solely due to *T_1_* effects, (b) Normalized *M* signal change with field strength which was calculated from fractional *Δ* *M* by multiplying the intrinsic SNR of *M_0_*, which is proportional to the field strength.

Based on the imaging parameters, theoretical calculation of the ASL perfusion signal as a function of field strength was carried out in two steps. The first step was to calculate the fraction of *ΔM* signal changes with field strength solely due to the *T_1_* effect (according to Eqs. [4] and [6]). The signal increase in the fraction of *ΔM* signal due to the *T_1_* increase as the field changes from 3T to 7T is 1.36 and 1.27 times for PASL and pCASL respectively (Fig. 2a). As the second step, we calculated the normalized *ΔM* signal by multiplying the result of step 1 with the intrinsic SNR of *M_0_*, which is proportional to the field strength (Fig. 2b). The relative SNR acquired by Turbo-FLASH for 7T pCASL, 7T PASL, 3T pCASL and 3T PASL is 3.7∶3.1∶1.3∶1, i.e., an approximate factor of 3 when performing ASL at 7T versus the same technique at 3T using Turbo-FLASH as the readout.

### Exp 1. Resting-state pCASL and PASL

**Figure 3:**
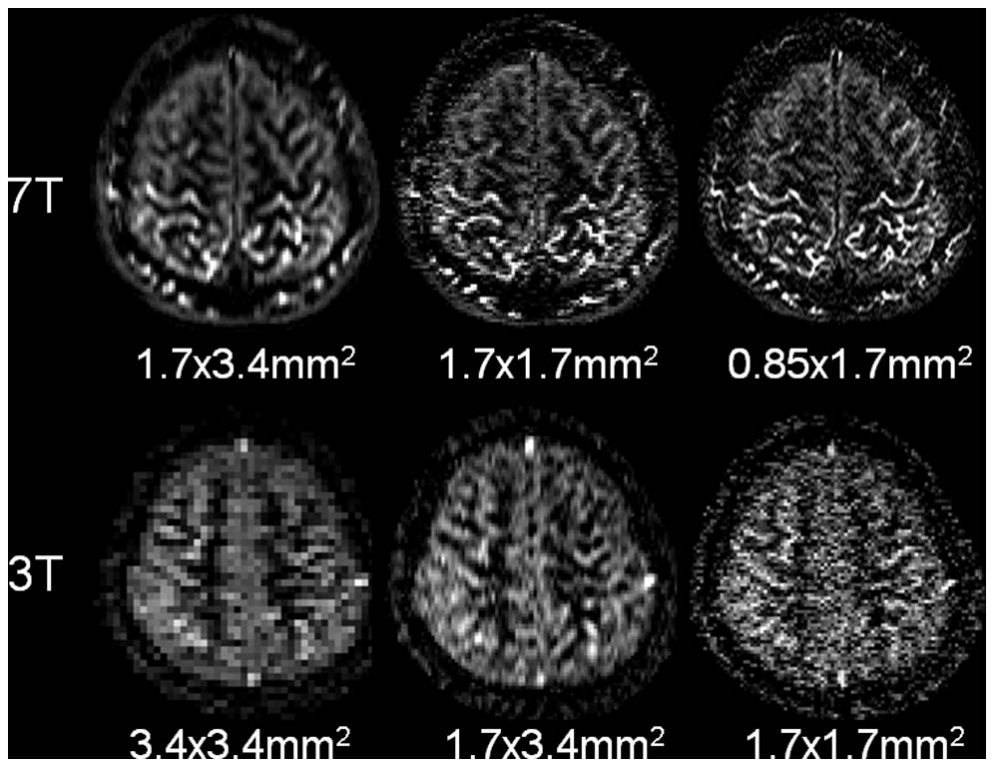
Pseudo-continuous ASL difference perfusion images (*Δ* *M*) at 7 T (top) and 3 T (bottom) with 3 different resolutions.

Figure 3 shows a direct comparison of Turbo-FLASH based pCASL perfusion images acquired at 7T and 3T with 3 spatial resolutions (1.7×3.4 mm^2^, 1.7×1.7 mm^2^ and 0.85×1.7 mm^2^ at 7T, 3.4×3.4 mm^2^, 1.7×3.4 mm^2^ and 1.7×1.7 mm^2^ at 3T). In terms of image quality, the perfusion images at 7T provide clearer delineation of cortical structures than those acquired at 3T. At 7T, the perfusion image quality does not degrade with higher resolution while 3T images appear noisy at the high resolution of 1.7×1.7 mm^2^. The measured mean SNR of pCASL was 4.21±0.59 at 7T compared to 1.12±0.20 at 3T for the identical resolution of 1.7×3.4 mm^2^ (i.e. 3.8 times SNR gain). The measured mean SNR of pCASL was 2.84±0.41 at 7T compared to 0.71±0.17 at 3T for the identical resolution of 1.7×1.7 mm^2^ (i.e. 4 times SNR gain) (Table 1). The SNR ratio of pCASL and PASL were 1.49 and 1.24 at 3T and 7T respectively. The temporal SNR of pCASL perfusion image series at 7T, however, was reduced by approximately 40% as compared to that at 3T (see Table 2).

**Table 1:** The spatial SNR at 7 T and 3 T.

Resolutions(mm^2^)	pCASL				PASL	
	3.4×3.4	1.7×3.4	1.7×1.7	0.85×1.7	3.4×3.4	1.7×3.4
3 T	1.71±0.27	1.12±0.20	0.71±0.17		1.15±0.08	
7 T		4.21±0.59	2.84±0.41	1.73±0.38		3.40±0.71

Average SNR of Turbo-FLASH based pCASL and PASL with different spatial resolutions at 7 T and 3 T.

**Table 2:** The temporal SNR at 7 Τ and 3 Τ.

Resolutions (mm^2^)	pCASL				PASL	
	3.4×3.4	1.7×3.4	1.7×1.7	0.85×1.7	3.4×3.4	1.7×3.4
3 Τ	12.4±2.4	10.0±1.5	10.5±2.1		16.7±2.8	
7 Τ		5.8±2.4	6.5±2.6	4.7±1.8		24.3±7.2

Average temporal SNR of Turbo-FLASH based pCASL and PASL with different spatial resolutions at 7 Τ and 3 Τ.

The labeling efficiency of pCASL at 7T was estimated by normalizing the mean CBF values to those acquired by PASL of the same subjects (mean ± SD = 54.90±1.40 ml/100 g/min across 5 subjects). Assuming an efficiency of 0.95 for PASL at 3T [22], this resulted in a mean PASL efficiency of 0.93±0.06 and pCASL efficiency of 0.64±0.06 at 7T.

As shown in Table 1, the SNR of 7T PASL (3.40±0.71) was approximately 3 times that of 3T PASL (1.15±0.08) even with a higher spatial resolution of 1.7×3.4 mm^2^. Furthermore, 7T PASL exhibited a higher level of temporal SNR (24.3±7.2) than its counterpart at 3T (16.7±2.8, see Table 2).

### Exp 2. fMRI of Motor Cortex Activation and Functional Connectivity using PASL

**Figure 4:**
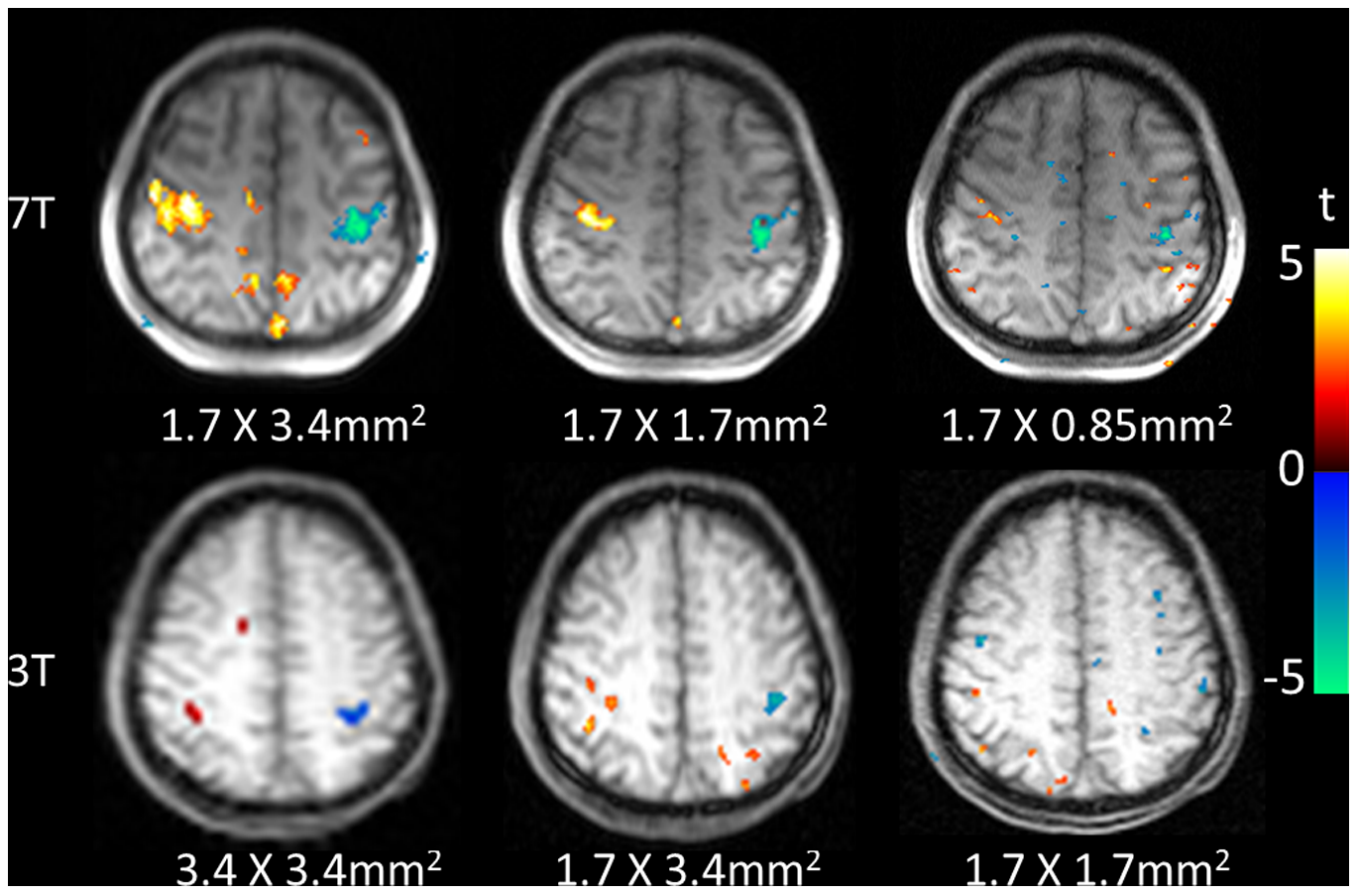
PASL perfusion fMRI results of finger tapping. Single-slice PASL perfusion fMRI results of alternating finger tapping overlaid on original Turbo-FLASH images with different resolutions at 7 Τ (top) and 3 Τ (bottom).

**Figure 5:**
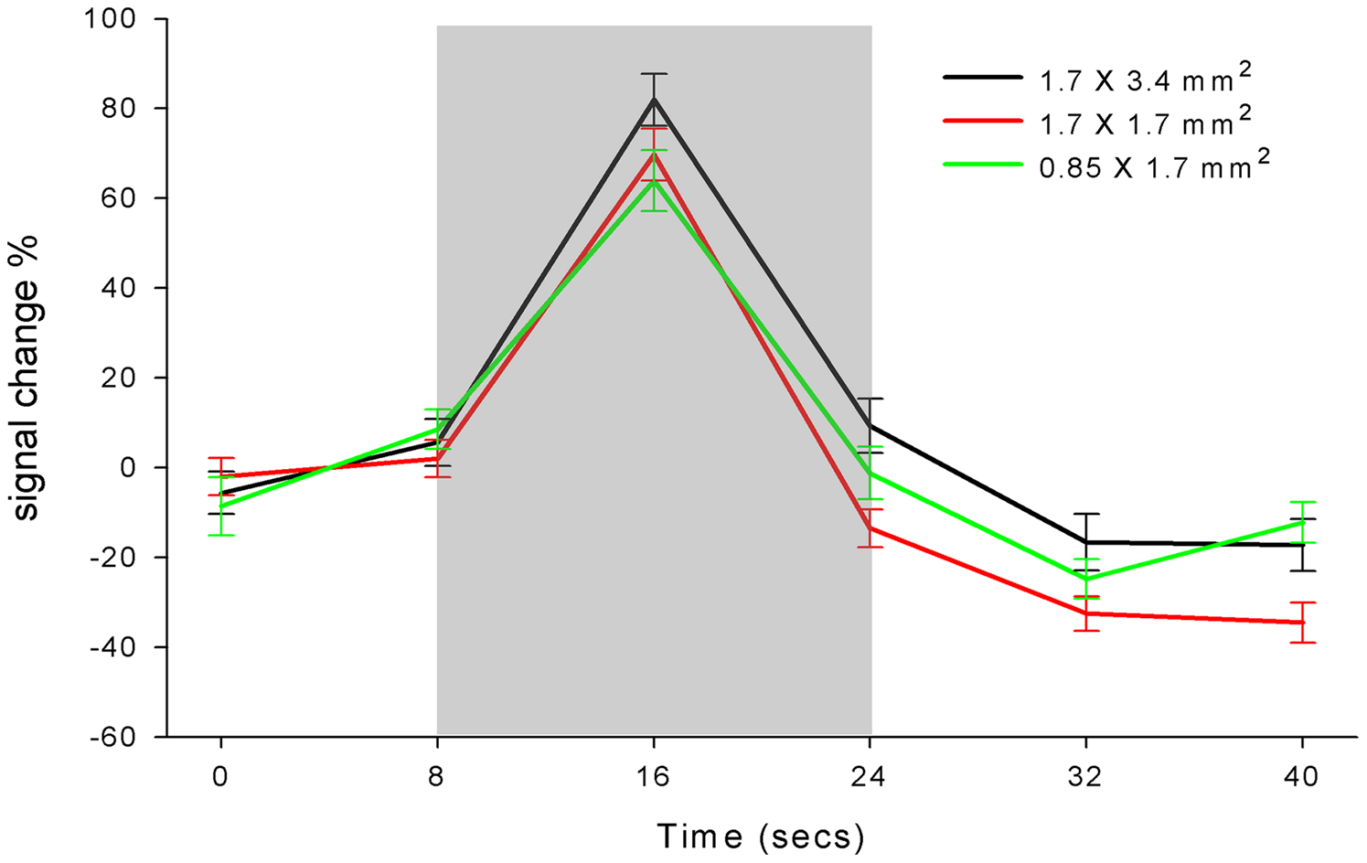
Time course of fMRI. Mean time course of perfusion fMRI scans with different resolutions at 7 Τ, gray column represents the time period of task activation.

Figure 4 shows t-statistic maps of motor cortex activation of a representative subject with 3 spatial resolutions at 3T and 7T. As shown in Figure 5, the mean peak-to-peak perfusion signal changes corresponding to 81.9±5.7, 69.8±5.8 and 64.0±6.8% for the resolution of 1.7×3.4 mm^2^, 1.7×1.7 mm^2^ and 0.85×1.7 mm^2^ at 7T, respectively. There were no significant differences between the 3 different resolutions (*p* = 0.084). The high resolution fMRI map precisely located primary motor cortex in the precentral gyrus at 7T, while 3T PASL failed to accurately locate the motor cortex when the spatial resolution was higher than 1.7×1.7 mm^2^.

**Figure 6:**
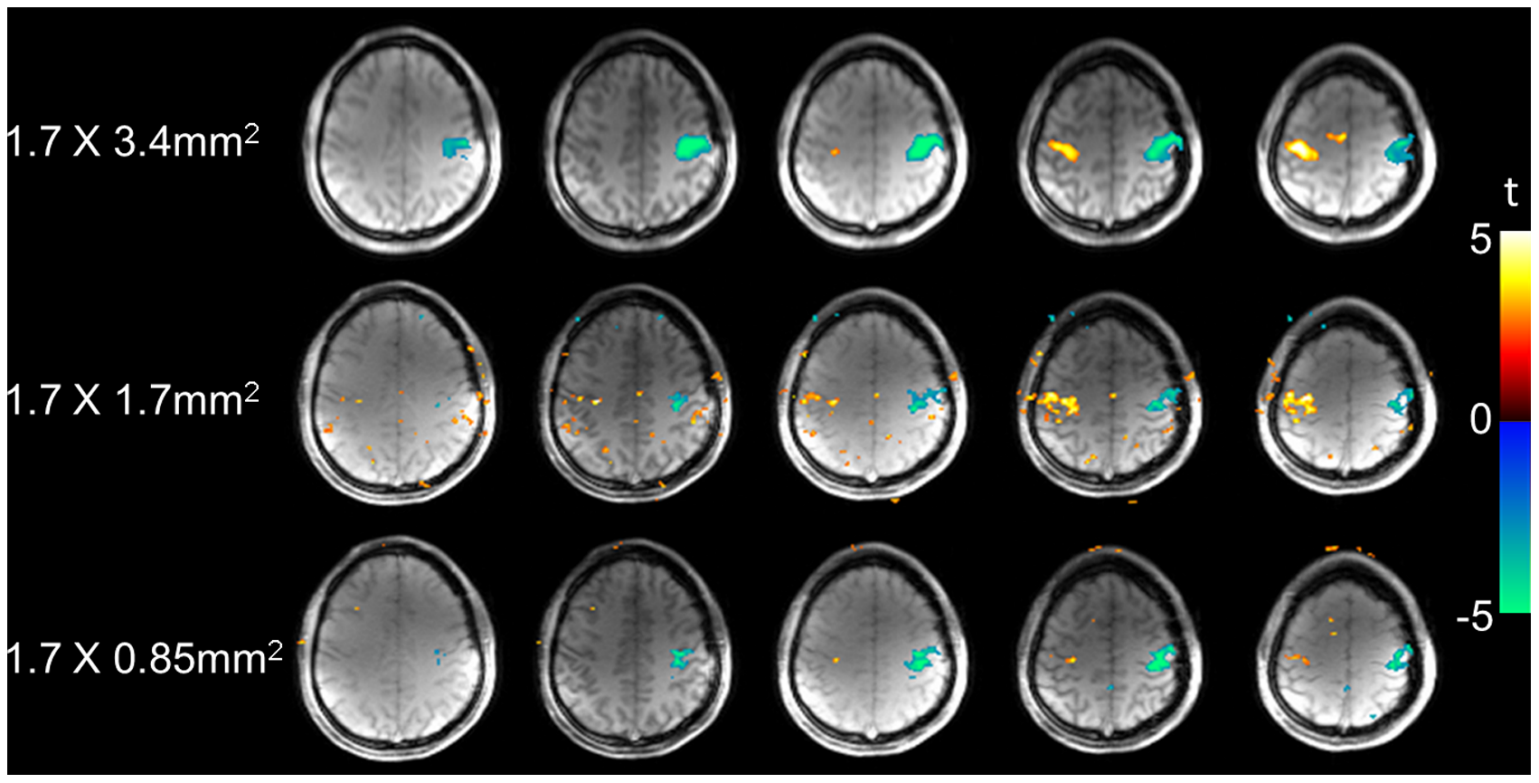
Multi-slice perfusion functional MRI maps. Multi-slice perfusion fMRI maps with different spatial resolutions using an alternating finger tapping paradigm at 7 Τ.

**Figure 7:**
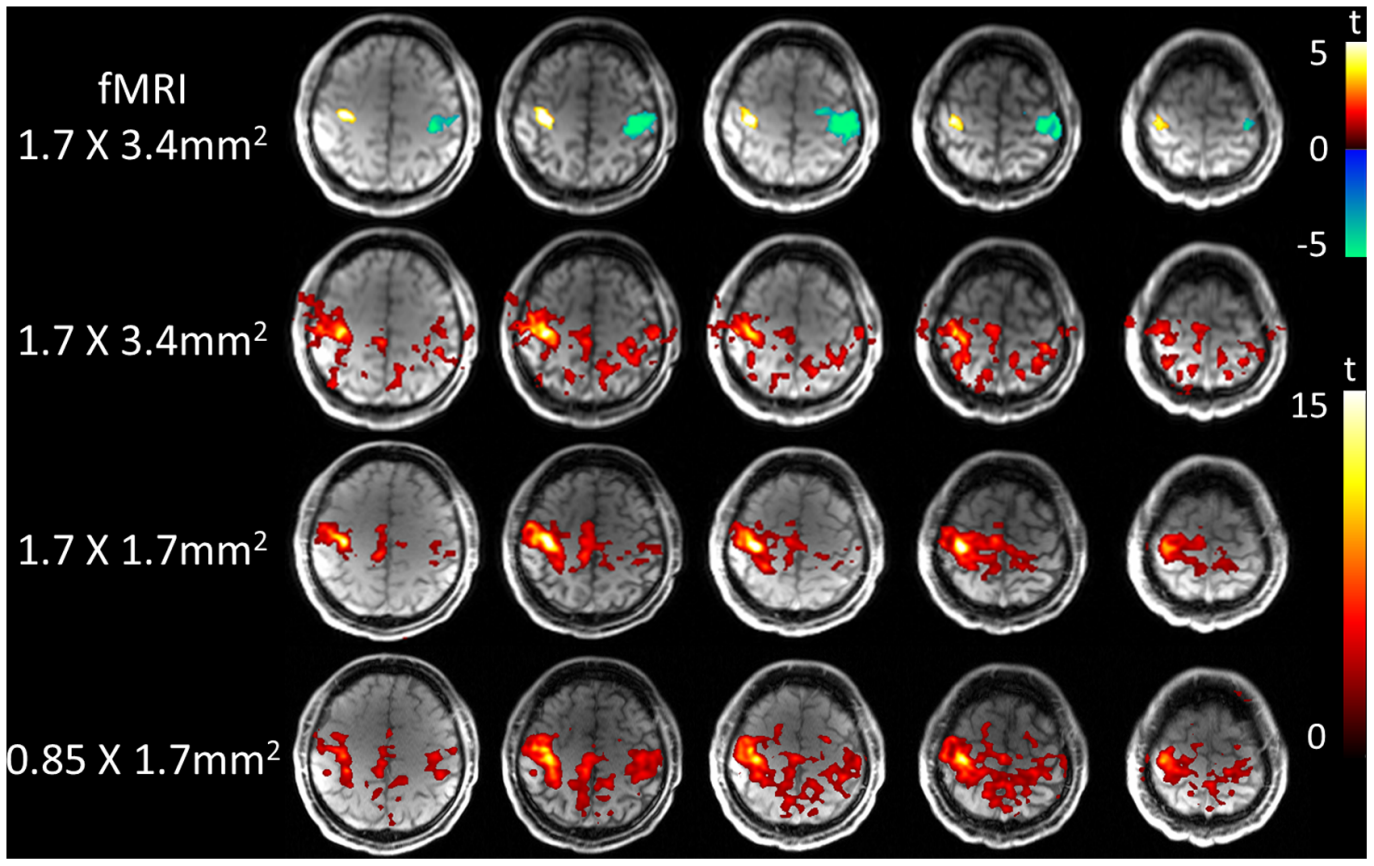
Functional connectivity maps. Multi-slice functional connectivity maps with different spatial resolutions generated using the left motor cortex as the seed based on resting state PASL scans at 7 Τ. The upper row shows task activation fMRI maps of alternating finger tapping.

Figure 6 displays the fMRI results of the finger-tapping task using multi-slice PASL with the spatial resolution of 1.7×3.4 mm^2^, 1.7×1.7 mm^2^ and 0.85×1.7 mm^2^ at 7 Τ. Figure 7 displays the functional connectivity maps generated using the left motor cortex as the seed, based on resting state PASL scans. As can be clearly seen in Fig. 7 and Table 3 of mean correlation coefficients, the right motor cortex was significantly correlated with left motor cortex, and vice versa. The supplemental motor area (SMA) was also significantly correlated with left and right motor cortices.

**Table 3:** Correlation analysis of functional connectivity.

Resolutions (mm^2^)	1.7×3.4	1.7×1.7	0.85×1.7
r (between left and right motor cortices)	0.7223	0.7322	0.8085
r (between SMA and bilateralmotor cortices)	0.8252	0.8829	0.7798

Pearson correlation coefficients (*r*) between left and right motor cortices, and between SMA and bilateral motor cortices at 7 Τ (p<0.001).

### Phantom Results

There was a 2.98 times SNR gain at 7 Τ compare with 3 Τ as measured on the TSE PD images. Accounting for the 2.33 times SNR gain from 3 to 7 Τ, this result suggests a 1.28 times SNR gain using the 24 channel Nova Medical coil compared with the default 12 channel coil.

## Discussions

The results presented above demonstrate the feasibility and benefits of Turbo-FLASH based ASL approaches at ultrahigh magnetic fields. Due to prolonged blood *T_1_* and higher intrinsic SNR, our theoretical simulations indicated an approximate factor of 3 in SNR gain by performing Turbo-FLASH based ASL at 7T relative to 3T. Our experimental results showed a SNR gain of 3.7 to 4.0 when comparing Turbo-FLASH based pCASL at 7T vs. 3T. Since there is an additional 1.28 times SNR gain by using the custom 24-ch (Nova Medical) receiver coil at 7T vs. the default 12-ch receiver coil at 3T, our experimental results match well with the theoretical prediction (i.e., 3×1.28 = 3.84 SNR gain). This high SNR gain allowed perfusion imaging with a high in-plane resolution of 0.85×1.7 mm^2^ at 7T. Turbo-FLASH is emerging as an appealing imaging approach at 7T, which has been successfully applied for structural, phase and susceptibility MRI [16], [17], [18]. The present study further demonstrates that it may be an ideal approach for perfusion MRI at ultrahigh fields.

The drawbacks of Turbo-FLASH, as compared to EPI, include relatively long scan time and potential saturation effects (on the label) of the RF pulse train. In the present study, each slice acquisition with a matrix size of 128×128 took 395 ms which limited the imaging coverage to a single or a few slices. In comparison, it generally takes less than 60ms to acquire a single slice using EPI. To demonstrate the intrinsic ASL signal gain at high fields, we did not apply partial k-space or parallel imaging which would have reduced the slice acquisition time by a factor of 2 to 4 at the cost of a lowered SNR [29], [30]. In future studies, partial k-space and/or parallel imaging should be carried out in conjunction with centric order sampling to improve the imaging speed of Turbo-FLASH.

In the present study, a single-compartment model was employed for perfusion quantification as well as for the simulation of Turbo-FLASH based ASL signal as a function of field strength. It is reasonable to assume that the labeled water stays in the vasculature rather than exchanging into brain tissue since the bolus duration was relatively short (700 ms) for both PASL and pCASL in our experiment, while the exchange time of labeled water is on the order of 1.5 s [31]. Using a two-compartment model including both vascular and tissue compartments [32], the potential error in Turbo-FLASH based PASL and pCASL signals is 2.0% and 0.3% at 7T compared to those calculated using a single-compartment model.

While Turbo-FLASH can improve the readout of ASL perfusion signals at 7T, the major challenge of implementing ASL at ultrahigh fields is inhomogeneities of B_0_ and B_1_ fields. The RF wavelength in human tissue at 7T is about 11 cm compared to 26cm at 3T, thus approaching the human head size. A local transmit coil is commonly used for RF-delivery to minimize the inhomogeneous B_1_-distribution, which is optimized in the cerebellum. However, for spin labeling at the base of the brain or close to the neck, B_1_ and B_0_ field inhomogeneities deteriorate which may significantly affect the labeling efficiency of both PASL and pCASL. In the present study, we attempted to minimize field inhomogeneity effects by placing both imaging and labeling regions on the brain cortex. The labeling plane of pCASL was 4.5 cm below the imaging slice through the motor cortex, while the gap between the tagging slab and imaging slice was 2 cm in PASL. The estimated PASL efficiency was 0.93±0.06 and pCASL efficiency was 0.64±0.06 at 7T, which is acceptable. In the future, several approaches may be applied for effective spin labeling at the base of the brain at ultrahigh fields. One is to place the iso-center of the main field at or close to the labeling region to minimize the effect of B_0_ inhomogeneity, as suggested by a recent pCASL study on mice at 11.75T [33]. Although the imaging volume may be off-center, the use of Turbo-FLASH readout is resistant to B_0_ inhomogeneity effects. Second, to improve B_1_ homogeneity at the labeling region, novel coil design may be applied with extended coverage in the neck region [34]. An alternative approach is to apply separate neck labeling coils which may also reduce the overall SAR level [35]. In addition, a phase-cycling approach may be applied to reduce phase error in pCASL at high fields [36].

The present study also demonstrated perfusion fMRI of motor cortex activation at 7T with an in-plane resolution as high as 0.85×1.7 mm^2^. In contrast, at 3T when the resolution was higher than 1.7×3.4 mm^2^, the SNR decreased significantly which caused spurious activation. The mean percentage of perfusion signal changes did not vary significantly between different spatial resolutions at 7T. This result appears in contradiction with the study by Pfeuffer et al. [9] that reported increased perfusion signal change with higher spatial resolution (>200% at the highest resolution of 0.9×0.9×1.5 mm^3^). The authors attributed their findings to significant partial volume effects in perfusion fMRI. There are several differences between the Pfeuffer study and our study. The original FAIR technique was used by Pfeuffer et al. with a delay of 1.5 s while QUIPSS II type FAIR was used in the present study with a tagging bolus of 700 ms and a post-labeling delay of 1 s (total delay of 1.7 s). Transit effects (intravascular signals) are not suppressed in FAIR whereas they are largely suppressed using QUIPSS II. Therefore, the high perfusion signal changes (>200%) observed by Pfeuffer et al. with the resolution of 0.9×0.9×1.5 mm^3^ may reflect flow increases in small vessels. It is worth noting that the reported perfusion signal changes at comparable spatial resolutions were similar between our study and Pfeuffer study. These results suggest that the observed fMRI activation in our study primarily reflects perfusion signal changes in capillaries and brain tissue. Given recent evidence that perfusion based fMRI may be more stable and offer higher spatial accuracy than BOLD fMRI [37], ultrahigh field ASL may provide a promising tool for fMRI studies.

Another recent trend in fMRI is functional connectivity within a number of brain networks during the resting state [38], [39], such as the default mode, visual, auditory and motor networks. Perfusion based fMRI has been attempted for detecting resting state functional connectivity, and several ASL studies reported functional connectivity of the default mode and motor networks [40], [41]. However, one challenge for perfusion based functional connectivity studies is the potential contamination of blood-oxygen level dependent (BOLD) contrast during ASL scans, although a temporal filtering algorithm has been proposed [42] to separate perfusion and BOLD effects. Another challenge is the relatively weak ASL signal that hampers the sensitivity and reliability for detecting functional connectivity within brain networks. Turbo-FLASH based ASL at 7 Τ may provide an ideal tool for perfusion based functional connectivity studies, given the approximately 4 times SNR gain compared to the same technique at 3 Τ. In addition, the use of Turbo-FLASH as the readout of ASL signals minimizes the susceptibility or BOLD effect. In future, further technical development is required to improve the imaging speed and coverage of Turbo-FLASH based ASL at 7 Τ so that it can be reliably applied for resting state functional connectivity studies.

In the present study, the temporal SNR of 7 Τ pCASL was 40% lower than that of 3 Τ pCASL. But the temporal SNR of 7 Τ PASL was approximately 50% greater than that of PASL at 3 Τ. The reason for the discrepancies between temporal SNR levels of pCASL and PASL at 3 Τ and 7 Τ is not well understood and may be related to different effects of physiological noise on ASL signals at 3 Τ and 7 Τ. Past studies have generally reported a higher level of temporal SNR using pCASL compared to PASL at 3 Τ, with EPI as the readout sequence [43]. In the present study, however, the label duration (700 ms) of pCASL was relatively short and the labeling efficiency may be affected by temporal variations of B_0_ and B_1_ fields due to field drifts, cardiac and respiratory effects at 7 Τ. With short adiabatic pulses, PASL may be less sensitive to potential variations of B_0_ and B_1_ fields compared to pCASL at 7 Τ. This is a hypothesis to be tested in future studies.

### Conclusion

Turbo-FLASH based ASL is a promising approach to maximize the SNR gain of 7T ASL, while minimizing field inhomogeneity effects and improving the spatial and temporal resolution of ultrahigh field ASL. It is feasible to apply multi-slice Turbo-FLASH based ASL for resting and task activation perfusion imaging at 7Τ. Approximately 4 times SNR gain is readily achievable at 7T compared to 3T, which opens the door to ultrahigh field fMRI with high spatiotemporal resolution as well as novel approaches to investigate the functional connectivity of brain networks.

## References

[1] Detre JA, Alsop DC (1999) Perfusion fMRI with arterial spin labeling (ASL). In: Moonen CTW, Bandettini PA, editors. Functional MRI. Heidelberg: Springer. 47–62.

[2] Detre JA, Wang J, Wang Z, Rao H (2009) Arterial spin-labeled perfusion MRI in basic and clinical neuroscience. Current Opinion in Neurology 22: 348–355. 19491678 10.1097/WCO.0b013e32832d9505

[3] Wong EC (1999) Potential and pitfalls of arterial spin labeling based perfusion imaging techniques for MRI. In: Moonen CTW, Bandettini PA, editors. Functional MRI. Heidelberg: Springer. 63–69.

[4] Wong EC, Cronin M, Wu W-C, Inglis B, Frank LR, et al (2006) Velocity-selective arterial spin labeling. Magnetic Resonance in Medicine 55: 1334–1341. 16700025 10.1002/mrm.20906

[5] Wang J, Alsop DC, Li L, Listerud J, Gonzalez-At JB, et al (2002) Comparison of quantitative perfusion imaging using arterial spin labeling at 1.5 and 4.0 Tesla. Magnetic Resonance in Medicine 48: 242–254. 12210932 10.1002/mrm.10211

[6] Teeuwisse WM, Webb AG, van Osch MJP (2010) Arterial spin labeling at ultra-high field: All that glitters is not gold. International Journal of Imaging Systems and Technology 20: 62–70.

[7] Rane SD, Gore JC (2013) Measurement of T1 of human arterial and venous blood at 7 Τ. Magnetic Resonance Imaging 31: 477–479. 23102945 10.1016/j.mri.2012.08.008 PMC3561475

[8] Kjølby BF, Østergaard L, Kiselev VG (2006) Theoretical model of intravascular paramagnetic tracers effect on tissue relaxation. Magnetic Resonance in Medicine 56: 187–197. 16724299 10.1002/mrm.20920

[9] Pfeuffer J, Adriany G, Shmuel A, Yacoub E, Van De Moortele P-F, et al (2002) Perfusion-based high-resolution functional imaging in the human brain at 7 Tesla. Magnetic Resonance in Medicine 47: 903–911. 11979569 10.1002/mrm.10154

[10] Gardener AG, Gowland PA, Francis ST (2009) Implementation of quantitative perfusion imaging using pulsed arterial spin labeling at ultra-high field. Magnetic Resonance in Medicine 61: 874–882. 19189295 10.1002/mrm.21796

[11] Luh W-M, Talagala SL, Li T-Q, Bandettini PA (2013) Pseudo-continuous arterial spin labeling at 7T for human brain: Estimation and correction for off-resonance effects using a Prescan. Magnetic Resonance in Medicine 69: 402–410. 22488568 10.1002/mrm.24266 PMC3402610

[12] Ziener CH, Kampf T, Melkus G, Jakob PM, Bauer WR (2007) Scaling laws for transverse relaxation times. Journal of Magnetic Resonance 184: 169–175. 17045825 10.1016/j.jmr.2006.09.018

[13] St Lawrence KS, Wang J (2005) Effects of the apparent transverse relaxation time on cerebral blood flow measurements obtained by arterial spin labeling. Magn Reson Med 53: 425–433. 15678532 10.1002/mrm.20364

[14] Günther M, Bock M, Schad LR (2001) Arterial spin labeling in combination with a look-locker sampling strategy: Inflow turbo-sampling EPI-FAIR (ITS-FAIR). Magnetic Resonance in Medicine 46: 974–984. 11675650 10.1002/mrm.1284

[15] Jahng G-H, Weiner MW, Schuff N (2007) Improved arterial spin labeling method: Applications for measurements of cerebral blood flow in human brain at high magnetic field MRI. Medical Physics 34: 4519–4525. 18072518 10.1118/1.2795675 PMC2443744

[16] Marques JP, Kober T, Krueger G, van der Zwaag W, Van de Moortele P-F, et al (2010) MP2RAGE, a self bias-field corrected sequence for improved segmentation and T1-mapping at high field. NeuroImage 49: 1271–1281. 19819338 10.1016/j.neuroimage.2009.10.002

[17] Lee J, Shmueli K, Fukunaga M, van Gelderen P, Merkle H, et al (2010) Sensitivity of MRI resonance frequency to the orientation of brain tissue microstructure. Proceedings of the National Academy of Sciences 107: 5130–5135. 10.1073/pnas.0910222107 PMC2841900 20202922

[18] Cho Z-H, Han J-Y, Hwang S-I, Kim D-s, Kim K-N, et al (2010) Quantitative analysis of the hippocampus using images obtained from 7.0T MRI. NeuroImage 49: 2134–2140. 19909820 10.1016/j.neuroimage.2009.11.002

[19] Bauer WR, Hiller KH, Roder F, Rommel E, Ertl G, et al (1996) Magnetization exchange in capillaries by microcirculation affects diffusion-controlled spin-relaxation: a model which describes the effect of perfusion on relaxation enhancement by intravascular contrast agents. Magn Reson Med 35: 43–55. 8771021 10.1002/mrm.1910350107

[20] Wong EC, Buxton RB, Frank LR (1998) Quantitative imaging of perfusion using a single subtraction (QUIPSS and QUIPSS II). Magnetic Resonance in Medicine 39: 702–708. 9581600 10.1002/mrm.1910390506

[21] Kim S-G (1995) Quantification of relative cerebral blood flow change by flow-sensitive alternating inversion recovery (FAIR) technique: Application to functional mapping. Magnetic Resonance in Medicine 34: 293–301. 7500865 10.1002/mrm.1910340303

[22] Wu W-C, Fernández-Seara M, Detre JA, Wehrli FW, Wang J (2007) A theoretical and experimental investigation of the tagging efficiency of pseudocontinuous arterial spin labeling. Magnetic Resonance in Medicine 58: 1020–1027. 17969096 10.1002/mrm.21403

[23] Buxton RB, Frank LR, Wong EC, Siewert B, Warach S, et al (1998) A general kinetic model for quantitative perfusion imaging with arterial spin labeling. Magnetic Resonance in Medicine 40: 383–396. 9727941 10.1002/mrm.1910400308

[24] Wang J, Licht DJ, Jahng G-H, Liu C-S, Rubin JT, et al (2003) Pediatric perfusion imaging using pulsed arterial spin labeling. Journal of Magnetic Resonance Imaging 18: 404–413. 14508776 10.1002/jmri.10372

[25] Balu N, Yarnykh VL, Chu B, Wang J, Hatsukami T, et al (2011) Carotid plaque assessment using fast 3D isotropic resolution black-blood MRI. Magnetic Resonance in Medicine 65: 627–637. 20941742 10.1002/mrm.22642 PMC3042490

[26] Wang J, Zhang Y, Wolf RL, Roc AC, Alsop DC, et al (2005) Amplitude-modulated Continuous Arterial Spin-labeling 3.0-T Perfusion MR Imaging with a Single Coil: Feasibility Study1. Radiology 235: 218–228. 15716390 10.1148/radiol.2351031663

[27] Golay X, Petersen ET, Hui F (2005) Pulsed star labeling of arterial regions (PULSAR): A robust regional perfusion technique for high field imaging. Magnetic Resonance in Medicine 53: 15–21. 15690497 10.1002/mrm.20338

[28] Dixon JE, Simpson A, Mistry N, Evangelou N, Morris PG (2013) Optimisation of T2*-weighted MRI for the detection of small veins in multiple sclerosis at 3 Τ and 7 Τ. European Journal of Radiology 82: 719–727. 22138119 10.1016/j.ejrad.2011.09.023

[29] Sodickson DK, Griswold MA, Jakob PM, Edelman RR, Manning WJ (1999) Signal-to-noise ratio and signal-to-noise efficiency in SMASH imaging. Magnetic Resonance in Medicine 41: 1009–1022. 10332885 10.1002/(sici)1522-2594(199905)41:5<1009::aid-mrm21>3.0.co;2-4

[30] Ohliger MA, Grant AK, Sodickson DK (2003) Ultimate intrinsic signal-to-noise ratio for parallel MRI: Electromagnetic field considerations. Magnetic Resonance in Medicine 50: 1018–1030. 14587013 10.1002/mrm.10597

[31] Wang J, Alsop DC, Song HK, Maldjian JA, Tang K, et al (2003) Arterial transit time imaging with flow encoding arterial spin tagging (FEAST). Magn Reson Med 50: 599–607. 12939768 10.1002/mrm.10559

[32] St. Lawrence KS, Owen D, Wang DJJ (2012) A two-stage approach for measuring vascular water exchange and arterial transit time by diffusion-weighted perfusion MRI. Magnetic Resonance in Medicine 67: 1275–1284. 21858870 10.1002/mrm.23104 PMC4066380

[33] Duhamel G, Tachrount M, Cozzone PJ, Alsop DC, Callot V (2011) Pseudocontinuous Arterial Spin Labeling (pCASL) at Very High Field (11.75T) for Mouse Brain Perfusion Imaging. In: Proceedings of the ISMRM 19th Annual Meeting. Montreal. 371.

[34] Teeuwisse WM, Webb AG, Van Osch MJP (2010) Whole Brain Pseudo Continuous ASL at 7 Τ Using a Single Coil for Imaging and Labeling. In: Proceedings of the ISMRM 18th Annual Meeting. Stockholm, Sweden. 1738.

[35] Talagala SL, Ye FQ, Ledden PJ, Chesnick S (2004) Whole-brain 3D perfusion MRI at 3.0T using CASL with a separate labeling coil. Magn Reson Med 52: 131–140. 15236376 10.1002/mrm.20124

[36] Jung Y, Wong EC, Liu TT (2010) Multiphase pseudocontinuous arterial spin labeling (MP-PCASL) for robust quantification of cerebral blood flow. Magnetic Resonance in Medicine 64: 799–810. 20578056 10.1002/mrm.22465

[37] Raoult H, Petr J, Bannier E, Stamm A, Gauvrit J-Y, et al (2011) Arterial spin labeling for motor activation mapping at 3 Τ with a 32-channel coil: Reproducibility and spatial accuracy in comparison with BOLD fMRI. NeuroImage 58: 157–167. 21689761 10.1016/j.neuroimage.2011.06.011

[38] Biswal B, Zerrin Yetkin F, Haughton VM, Hyde JS (1995) Functional connectivity in the motor cortex of resting human brain using echo-planar mri. Magnetic Resonance in Medicine 34: 537–541. 8524021 10.1002/mrm.1910340409

[39] Fox MD, Raichle ME (2007) Spontaneous fluctuations in brain activity observed with functional magnetic resonance imaging. Nat Rev Neurosci 8: 700–711. 17704812 10.1038/nrn2201

[40] Zou Q, Wu CW, Stein EA, Zang Y, Yang Y (2009) Static and dynamic characteristics of cerebral blood flow during the resting state. NeuroImage 48: 515–524. 19607928 10.1016/j.neuroimage.2009.07.006 PMC2739419

[41] Biswal BB, Kylen JV, Hyde JS (1997) Simultaneous assessment of flow and BOLD signals in resting-state functional connectivity maps. NMR in Biomedicine 10: 165–170. 9430343 10.1002/(sici)1099-1492(199706/08)10:4/5<165::aid-nbm454>3.0.co;2-7

[42] Chuang K-H, van Gelderen P, Merkle H, Bodurka J, Ikonomidou VN, et al (2008) Mapping resting-state functional connectivity using perfusion MRI. NeuroImage 40: 1595–1605. 18314354 10.1016/j.neuroimage.2008.01.006 PMC2435272

[43] Wu WC, Edlow BL, Elliot MA, Wang J, Detre JA (2009) Physiological modulations in arterial spin labeling perfusion magnetic resonance imaging. IEEE Trans Med Imaging 28: 703–709. 19150788 10.1109/TMI.2008.2012020

